# Bioactive Chalcone-Loaded Mesoporous Silica KIT-6 Nanocarrier: A Promising Strategy for Inflammation and Pain Management in Zebrafish

**DOI:** 10.3390/pharmaceutics17080981

**Published:** 2025-07-30

**Authors:** Maria Kueirislene Amâncio Ferreira, Francisco Rogenio Silva Mendes, Emmanuel Silva Marinho, Roberto Lima de Albuquerque, Jesyka Macedo Guedes, Izabell Maria Martins Teixeira, Ramon Róseo Paula Pessoa Bezerra de Menezes, Vinicius Patricio Santos Caldeira, Anne Gabriella Dias Santos, Marisa Jádna Silva Frederico, Antônio César Honorato Barreto, Inês Domingues, Tigressa Helena Soares Rodrigues, Jane Eire Silva Alencar de Menezes, Hélcio Silva dos Santos

**Affiliations:** 1 Postgraduate Program in Natural Sciences, Ceará State University, Fortaleza 60714-903, Brazil; kueirislene@hotmail.com (M.K.A.F.); rogenio.mendes@uece.br (F.R.S.M.); emmanuel.marinho@uece.br (E.S.M.); limarob2017@gmail.com (R.L.d.A.); jane.menezes@uece.br (J.E.S.A.d.M.); 2 Center for Exact Sciences and Technology, Vale do Acaraú University, Sobral 62040-370, Brazil; jesyka.mg@gmail.com (J.M.G.); tigressa_helena@uvanet.br (T.H.S.R.); 3 Department of Clinical and Toxicological Analysis, Federal University of Ceará, Fortaleza 60430-160, Brazil; izabellmaria@alu.ufc.br (I.M.M.T.); ramonppessoa@ufc.br (R.R.P.P.B.d.M.); 4 Laboratory of Catalysis, Environment and Materials, Department of Chemistry, Rio Grande do Norte State University, Mossoró 59610-210, Brazil; viniciuscaldeira@uern.br (V.P.S.C.);; 5 Department of Pharmacology and Physiology, Center for Drug Research and Development, School of Medicine, Federal University of Ceará, Fortaleza 60430-275, Brazil; marisafrederico@ufc.br; 6 Department of Physics, Federal University of Ceara, Fortaleza 60020-181, Brazil; 7 Center for Environmental and Marine Studies (CESAM), Department of Biology, University of Aveiro, Santiago University Campus, 3810-193 Aveiro, Portugal; inesd@ua.pt

**Keywords:** dibenzylideneacetone, controlled drug delivery, KIT-6, anti-inflammatory activity, antinociceptive activity, zebrafish

## Abstract

**Background/Objectives**: The incorporation of bioactive molecules into mesoporous carriers is a promising strategy to improve stability, solubility, and therapeutic efficacy. In this study, we report for the first time the encapsulation of the synthetic chalcone 4-Cl into KIT-6 mesoporous silica and evaluate its cytotoxicity, toxicological profile, and pharmacological activities (antinociceptive, anti-inflammatory, and anxiolytic) using an in vivo zebrafish (Danio rerio) model. **Methods**: Zebrafish were orally dosed with 4-Cl, 4-Cl/KIT-6, or KIT-6 (4, 20, 40 mg/kg) and mortality was recorded for 96 h. For analgesia, zebrafish pretreated with 4-Cl, 4-Cl/KIT-6, KIT-6, or morphine received a tail stimulus (0.1% formalin). Locomotor activity (quadrant crossings) was monitored for 30 min to assess analgesia (neurogenic: 0–5 min; inflammatory: 15–30 min). For inflammation, abdominal edema and weight gain were assessed 4 h after intraperitoneal carrageenan (1.5%). Zebrafish (*n* = 6/group) received 4-Cl, 4-Cl/KIT-6, or KIT-6 (4, 20, 40 mg/kg, p.o.). Controls received ibuprofen (100 mg/kg, p.o.) or 3% DMSO. Weight was measured hourly for 4 h post-carrageenan (difference between baseline and hourly weights). **Results**: Physicochemical characterizations confirmed successful encapsulation without compromising the ordered structure of KIT-6, as evidenced by a significant reduction in surface area and pore volume, indicating efficient drug incorporation. In vivo assays demonstrated that the 4-Cl/KIT-6 formulation maintained the pharmacological activities of the free chalcone, reduced toxicity, and, notably, revealed a significant anxiolytic effect for the first time. **Conclusions**: These findings highlight KIT-6 as a promising platform for chalcone delivery systems and provide a solid basis for future preclinical investigations.

## 1. Introduction

The development of effective treatments has always been one of the main priorities in medicine, initially driven by the use of bioactive compounds derived from plants. Over time, this traditional knowledge laid the foundation for the development of increasingly precise and controlled synthetic drugs [1,2]. With advances in modern pharmacology, new methodologies have emerged that enable a better understanding of drug interactions and the enhancement of therapeutic efficacy [3].

Among the most prevalent clinical conditions requiring pharmacological intervention are pain and inflammatory processes, both of which are often associated with significant functional limitations [4]. Both are essential yet complex physiological mechanisms. Pain arises from the activation of sensory receptors in response to noxious stimuli, while inflammation aims to repair damaged tissue and is characterized by redness, heat, swelling, pain, and loss of function [5,6,7].

However, conventional therapies used to manage these symptoms still face important challenges, such as low specificity, short duration of action, and adverse side effects—including gastrointestinal, hepatic, renal, and cardiovascular toxicity [8,9,10]. These limitations highlight the need for technologies capable of providing greater selectivity and controlled drug release, thereby reducing risks and improving therapeutic outcomes [11,12].

In this context, nanotechnology applied to controlled drug delivery has gained prominence by offering solutions to problems related to stability, solubility, and toxicity, enabling higher efficacy at lower doses [13,14,15]. Among the strategies investigated, the encapsulation of active molecules stands out as one of the most promising approaches, as it protects compounds from degradation, modulates their release kinetics, and increases their bioavailability [16,17,18,19].

Most reported formulations employ polymeric nanoparticles, liposomes, or cyclodextrin complexes, which often present limitations such as low stability, inefficient drug encapsulation, and rapid drug release. In contrast, mesoporous silica nanoparticles (MSNs), particularly materials such as MCM-41, SBA-15, SBA-16, and KIT-6, have demonstrated superior advantages, including high surface area, tunable porosity, thermal and chemical stability, and the ability for surface functionalization [20,21,22,23]. Among these, KIT-6 is especially attractive due to its highly ordered three-dimensional cubic symmetry (Ia3d), interconnected mesoporous channels, and large pore volume, which enable the incorporation of bulky molecules and promote efficient drug loading and controlled release. These features, combined with its chemical stability and reported biocompatibility, make KIT-6 an excellent platform for advanced drug delivery systems [24].

Chalcones represent a class of compounds with broad pharmacological potential. Composed of two aromatic rings linked by an α,β-unsaturated carbon chain, these molecules exhibit several biological activities, including anti-inflammatory, antimicrobial, antioxidant, and anxiolytic properties [25,26]. Among them, the chalcone (1E,4E)-1,5-bis (4-chlorophenyl)penta-1,4-dien-3-one (4-Cl), a dibenzylideneacetone derivative functionalized with two chlorine atoms, stands out. Its trans configuration and planar structure promote interactions with biological targets, contributing to its pharmacological activity. However, its low water solubility and susceptibility to degradation limit its direct therapeutic application [27]. Encapsulating this molecule in porous matrices such as KIT-6 silica may represent an effective strategy to overcome these limitations.

To evaluate the effects of bioactive compounds on inflammation and pain, the zebrafish (*Danio rerio*) has been widely used as an animal model. This organism offers several advantages, such as easy handling, high reproducibility, genetic similarity to humans, and a rapid and sensitive pharmacological response [28].

The overlap of mediators involved in these processes justifies the search for new molecules with anti-inflammatory and analgesic activity, particularly when combined with advanced drug delivery technologies.

Therefore, the objective of this study was to investigate the potential of mesoporous silica KIT-6 as a delivery system for the chalcone 4-cloro to evaluate its cytotoxic, toxicological, antinociceptive, and anti-inflammatory effects in an experimental zebrafish (*Danio rerio*) model.

## 2. Materials and Methods

### 2.1. Synthesis of the KIT-6 Mesoporous Material

The synthesis of the mesoporous material KIT-6 was carried out under acidic conditions using the hydrothermal method, as described by Kleitz et al. [29], following the molar ratio: 1 TEOS: 0.017 P123: 1.83 HCl: 195 H_2_O: 1.31 BuOH. To obtain 200 g of gel, 4.9 g of the non-ionic triblock copolymer Pluronic P123 (EO_20_PO_70_EO_20_, Pluronic P123 (EO20PO70EO20, Mw 5800, Sigma-Aldrich, St. Louis, MO, USA)), 176.4 mL of distilled water, and 7.6 mL of concentrated hydrochloric acid (37%, Dinâmica, Indaiatuba, SP, Brazil) were initially mixed. The mixture was stirred for 6 h at 30 °C. Then, 6 mL of *n*-butanol (≥99.4%, Vetec Química Fina Ltda., Duque de Caxias, RJ, Brazil) were added, and stirring continued for an additional hour at the same temperature. After this step, 11.4 mL of tetraethyl orthosilicate (TEOS, ≥98%, Sigma-Aldrich, St. Louis, MO, USA) were added as the silica source. The resulting mixture was stirred under the same conditions and subsequently aged in a Teflon-lined autoclave at 100 °C for 24 h under static hydrothermal conditions. The solid product was cooled, filtered, washed with a 2% alcoholic HCl solution, and dried at 100 °C for 12 h. Finally, the material was calcined in a muffle furnace at 550 °C for 6 h under an air atmosphere, with a heating rate of 10 °C per minute.

### 2.2. Encapsulation of 4-Chloro-Chalcone Within KIT-6

The encapsulation of 4-chloro-chalcone into mesoporous silica KIT-6 was carried out using the solvent evaporation method, adapted from Sayed et al. [30]. Initially, previously calcined KIT-6 (0.2053 g) was dispersed in 10 mL of ethanol (Vetec Química Fina Ltda., Duque de Caxias, RJ, Brazil) under magnetic stirring for 30 min to ensure the formation of a homogeneous suspension. Subsequently, 4-chloro-chalcone (0.2050 g, approximately 0.844 mmol) was added to the suspension, and the mixture was stirred continuously in a water bath at 40 °C for 48 h to promote the diffusion and adsorption of the compound into the porous matrix. After the incubation period, ethanol was removed under reduced pressure using a rotary evaporator at 60 °C. The resulting solid was further dried in an oven at 70 °C for 12 h to eliminate any residual solvent traces. The final material was stored in a sealed container and protected from light until further physicochemical characterization and biological evaluation.

### 2.3. Characterization

X-ray diffraction (XRD, Rigaku MiniFlex II diffractometer, Rigaku Corporation, Tokyo, Japan; λ = 1.5418 Å), operating at 30 kV and 15 mA. High-angle data were collected over a 2θ range of 5–90°, with a step size of 0.02° and 1 s per step. Low-angle measurements were performed from 0.5° to 3° (2θ), with a step size of 0.005° and 0.4 s per step. Thermogravimetric analysis (TGA), coupled with energy-dispersive X-ray spectroscopy (EDS), was conducted on a (Netzsch STA 449 F3 Jupiter thermoanalyzer (NETZSCH-Gerätebau GmbH, Selb, Germany)under a nitrogen flow of 60 mL·min^−1^, with a heating rate of 10 °C·min^−1^ from 30 to 900 °C, using alumina crucibles. Nitrogen adsorption–desorption isotherms were measured at 77 K using a ASAP 2020 adsorption analyzer (Micromeritics Instrument Corp., Norcross, GA, USA). Prior to analysis, samples were degassed at 170 °C for 10 h. Surface area was calculated using the BET method; pore volume and average pore diameter were estimated using the BJH method based on the desorption branch of the isotherms at a relative pressure (*p*/*p*_0_) of 0.98. FTIR spectra were obtained using a Bruker Alpha II spectrometer (Bruker Corporation, Billerica, MA, USA), employing the KBr pellet method in the range of 4000–400 cm^−1^, with 4 cm^−1^ resolution and 32 scans. Morphological analysis was performed by scanning electron microscopy (SEM) using a JEOL JSM-6390LV (JEOL Ltd., Tokyo, Japan). Zeta potential was measured from composite pellet suspensions prepared by dispersing the pellets in ultrapure water (for 4-Cl/KIT-6 and KIT-6) or ethanol (for 4-Cl), followed by analysis using a Zetasizer Nano ZS90 (Malvern Instruments Ltd., Malvern, UK).

### 2.4. Synthesis of Dibenzoylacetone (1E,4E)-1,5-Bis(4-Chlorophenyl)Penta-1,4-Dien-3-One (4-Cl)

**Figure 1:**
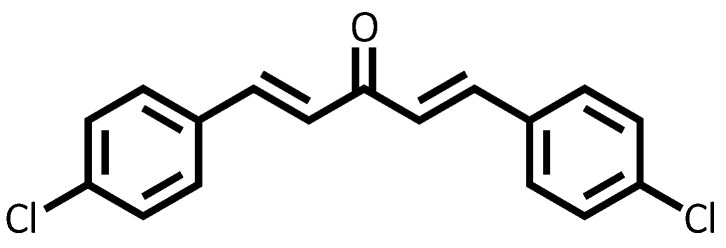
Structures of synthesized dibenzalacetone 4-Cl.

The oil phase consisted of chalcone, dimethyl sulfoxide (DMSO), Tween 80, and soybean vegetable oil (Figure 1). For the synthesis of dibenzalacetone, 0.5 mL of acetone and 1 g of NaOH were added to a 1:1 water–ethanol mixture. Then, 0.9 mL of benzaldehyde was added to this solution and stirred at 300 rpm in a magnetic stirrer for 30 min. Yellow crystals of dibenzalacetone were obtained, filtered under vacuum, washed with cold distilled water, and recrystallized with ethanol, dry at room temperature, for further analysis of characterization and biological activities [31].

### 2.5. Assessment of Biological Activities

#### Animals

For the in vivo assays, wild-type zebrafish (short-fin phenotype), of both sexes, aged 60–90 days, measuring approximately 3.5 ± 0.5 cm in length and weighing 0.4 ± 0.1 g, were used. The animals were obtained from a certified supplier (Agroquímica: Comércio de Produtos Veterinários LTDA, Fortaleza, Ceará, Brazil). Prior to testing, fish were acclimatized for 24 h in glass aquaria (30 × 20 × 15 cm) containing dechlorinated water (ProtecPlus^®,^ Labcon, São Paulo, SP, Brazil) under aeration with submerged filters, maintained at 25 °C and pH 7.0. Animals were fed commercial feed ad libitum up to 24 h before the experiments. For treatments, zebrafish were randomly assigned to groups (*n* = 6 per group) and received intraperitoneal (i.p.) injections of 4-Cl (4, 20, or 40 mg/kg; 20 µL) or vehicle (3% Dimethyl sulfoxide (DMSO, Sigma-Aldrich, St. Louis, MO, USA); 20 μL). For the 4-Cl/KIT-6 system, the administered dose was calculated based on the drug loading (34%) determined by thermogravimetric analysis (Section 4.1). The total mass of 4-Cl/KIT-6 administered was adjusted to deliver an equivalent amount of 4-Cl (4, 20, or 40 mg/kg) as used in the free compound groups, ensuring dose equivalence between treatments. Additionally, all animals were exposed by immersion in 1% ethanol for 20 min. Injections were performed using 0.5 mL insulin syringes (UltraFine^®^ BD, Becton, Dickinson and Company, Franklin Lakes, NJ, USA) fitted with 30 G needles [32]. After the experimental procedures, fish were euthanized by immersion in cold water (2–4 °C) until opercular movements ceased. All procedures were conducted in compliance with the Ethics Committee on Animal Use of the State University of Ceará (CEUA-UECE), under protocol No. 04983945/2021.

### 2.6. Assessment of Toxicological Effects

#### 2.6.1. Cytotoxicity

Substances were initially dissolved in sterile dimethyl sulfoxide (DMSO). The working concentrations of 4-Cl, 4-Cl/KIT-6, and KIT-6 (ranging from 250 to 15.6 µm) were prepared by serial dilution in sterile phosphate-buffered saline (PBS: NaCl 137 mm, KCl 2.7 mm, KH_2_PO_4_ 1.47 mm, Na_2_HPO_4_ 8.1 mm; pH 7.4), ensuring that the final DMSO content in the experimental wells did not exceed 0.5% (vehicle control). For in vivo administration, the 4-Cl/KIT-6 formulation was dispersed in PBS using mechanical homogenization immediately prior to treatment. The free 4-Cl compound was dissolved in DMSO (2%) and subsequently diluted in PBS to obtain the required concentrations. All dispersions were freshly prepared and visually inspected to ensure homogeneity, with no visible signs of sedimentation during the experimental period.

PC12 cells (ATCC CRL-1721; rat adrenal pheochromocytoma) were seeded in 96-well plates at a density of 1 × 10^5^ cells/mL (200 μL per well) in Dulbecco’s modified Eagle medium (DMEM) supplemented with 10% fetal bovine serum (FBS). Following overnight incubation at 37 °C in a 5% CO_2_ atmosphere, cells were exposed to treatments for 24 h and then subjected to the MTT reduction assay [33]. After treatment, cells were washed with sterile PBS, and MTT solution (0.25 mg/mL) was added to each well. After 4 h of incubation, the resulting formazan crystals were solubilized with DMSO, and absorbance was measured at 570 nm to calculate cell viability (%).

#### 2.6.2. Acute Toxicity

The animals received oral (v.o) treatments with the samples 4-Cl, 4-Cl/KIT-6, and KIT-6 at doses of 4, 20, and 40 mg/kg. Thirty minutes after administration, the animals were kept under continuous observation for a period of 96 h, to monitor the mortality rate [34]. Every 24 h, the number of deaths in each experimental group was recorded. The mean lethal dose (LD_50_), defined as the dose required to induce the death of 50% of exposed individuals, was determined using the trimmed Spearman–Karber statistical method, with a 95% confidence interval.

### 2.7. Formalin-Induced Nociceptive Behavior

The groups of animals were pretreated orally with 4-Cl, 4-Cl/KIT-6, KIT-6, and morphine, and after 1 h, they received treatment with a noxious stimulus (i.m. in the tail), induced by formalin (cation channel agonist with transient receptor potential, subfamily A, member 1 [TRPA1] neuropathic and neurogenic phase; 0.1%/5.0 µL). After formalin application, each animal was individually placed in a Petri dish containing quadrants, with aquarium water. Locomotor activity was monitored for 30 min, recording the number of times the fish crossed each quadrant. Analgesia was evaluated based on the locomotor activity of the animals, counting the number of crossings performed in the open field test in the Petri dish. The increase in locomotor activity reflects the analgesic effect, calculated in the neurogenic (0–5 min) and inflammatory (15–30 min) phases [35].

### 2.8. Anti-Inflammatory Activity

#### Induction of Abdominal Edema by Carrageenan

For the inflammation assay, abdominal edema and body weight gain were evaluated following the induction of inflammation with 1.5% carrageenan administered intraperitoneally for 4 h. Initially, untreated zebrafish were identified and weighed, and then randomly assigned into groups (*n* = 6 each). Three experimental groups received 4-Cl, 4-Cl/KIT-6, or KIT-6 at doses of 4, 20, or 40 mg/kg (p.o.). Additional groups were treated with ibuprofen (100 mg/kg, p.o.) as a positive control and 3% DMSO as a negative control. After a 1 h acclimation period, carrageenan (1.5%) was administered intraperitoneally to all animals, followed by another 1 h resting period. Subsequently, fish were weighed at hourly intervals for 4 consecutive hours. The results were expressed as the difference between the baseline weight (prior to carrageenan administration) and the weight recorded at each time point after induction, up to the final 4 h measurement [36].

### 2.9. Statistical Analysis

For bioassays with the zebrafish model, data were analyzed using Graphpad Prism software version 8.0. After confirming normal distribution and homogeneity of data, differences between groups were submitted to analysis of variance (one-way ANOVA and two-way ANOVA) in experiments with antagonists, followed by Tukey’s test. The level of statistical significance was set at 5% (*p* < 0.05). Cytotoxicity tests were performed in triplicate (*n* = 3). Results were expressed as mean ± standard error mean (SEM). Experimental groups were compared by one-way ANOVA (Dunnett’s post-test), with * *p* < 0.05 as the significance criterion.

## 3. Results

### 3.1. Structural Data of Chalcone 4-Cl

In the ^1^H NMR spectrum, two characteristic doublets appear at δ 7.04 ppm and 7.69 ppm with a coupling constant of 15.9 Hz, corresponding to the α and β protons of the enone system. The magnitude of the coupling constant is consistent with an E configuration of the double bond. The aromatic region shows signals at δ 7.40 ppm and 7.55 ppm, both as doublets with J = 8.0 Hz, which are attributed to the protons at positions 2/6 and 3/5, respectively. The ^13^C NMR spectrum displays a signal at δ 188.3 ppm, corresponding to the carbon of the α,β-unsaturated carbonyl group. Typically, saturated ketones resonate around δ 203.8 ppm; however, the observed upfield shift is likely due to delocalization of electron density across the conjugated system, involving the benzene ring and the double bond, which reduces the electron-withdrawing effect on the carbonyl carbon. The α and β olefinic carbons resonate at δ 125.7 and 142.0 ppm, respectively. Signals at δ 136.5, 133.2, 129.5, and 129.3 ppm are assigned to the aromatic carbons (C-4, C-1, C-3/5, and C-2/6), as detailed in Table 1.

### 3.2. Characterization

**Figure 2:**
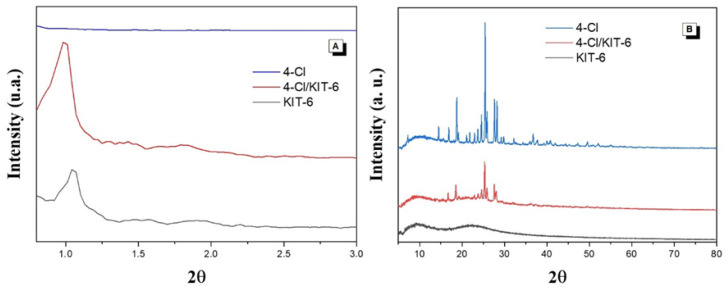
XRD: (**A**) Low-angle and (**B**) high-angle patterns of KIT-6, 4-Cl, and 4-Cl/KIT-6 materials.

Figure 2A shows the low-angle X-ray diffraction patterns of KIT-6 before and after encapsulation of the dichloro-chalcone (4-Cl). Three characteristic peaks associated with the Miller indices (211), (220), and (332) are observed, indicating the presence of an ordered mesoporous structure. Figure 2B displays the diffraction pattern of pure 4-Cl, with intense peaks at 2θ = 16.8°, 18.6°, 24.5°, 25.5°, 27.4°, and 28.2°, demonstrating its high crystallinity.

**Figure 3:**
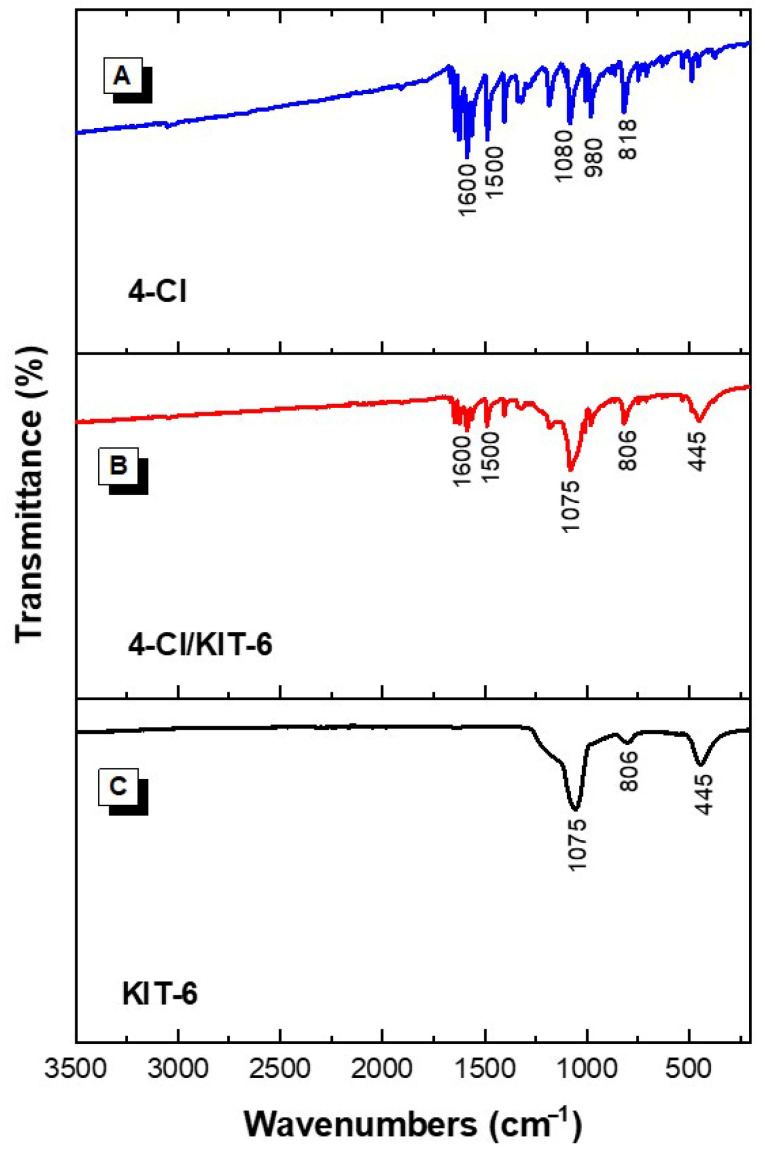
FTIR spectra of the samples: (**A**) 4-Cl, (**B**) 4-Cl/KIT-6, and (**C**) KIT-6.

The spectra obtained by Fourier-transform infrared spectroscopy (FTIR) analysis are shown in Figure 3, corresponding to 4-Cl chalcone, the KIT-6, and the encapsulated material 4-Cl/KIT-6.

**Figure 4:**
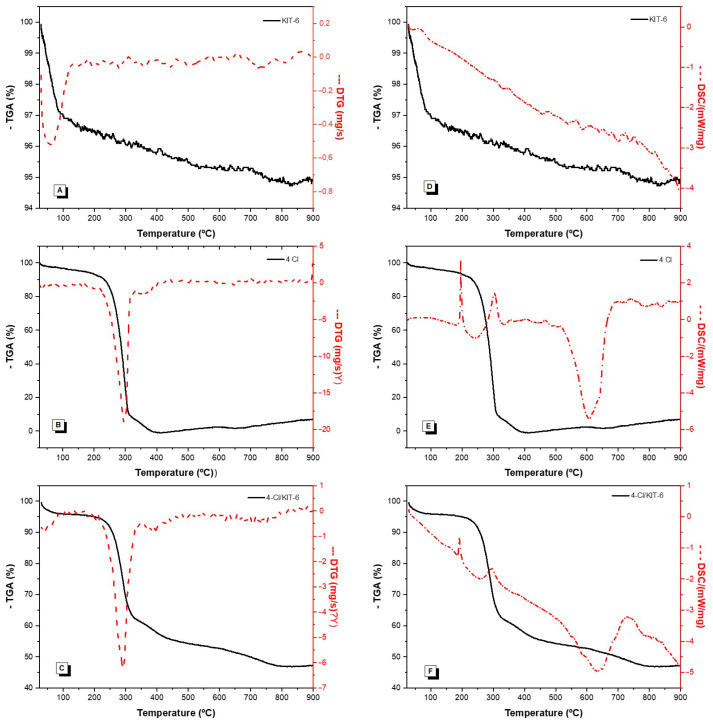
Thermal profiles of KIT-6, 4-Cl chalcone, and 4-Cl/KIT-6 samples obtained by thermogravimetric analysis (TGA/DTG) and differential scanning calorimetry (DSC). (**A**) TGA/DTG curve of KIT-6; (**B**) TGA/DTG curve of 4-Cl chalcone; (**C**) TGA/DTG curve of 4-Cl/KIT-6; (**D**) DSC curve of KIT-6; (**E**) DSC curve of 4-Cl chalcone; and (**F**) DSC curve of 4-Cl/KIT-6. TG curves are shown as solid lines and DSC curves as dashed lines.

Figure 4 shows the TGA/DTGA and DSC results of the analyzed samples, allowing the evaluation of the thermal profile and stability of the materials. The corresponding mass loss values from these analyses are summarized in Table 2.

**Figure 5:**
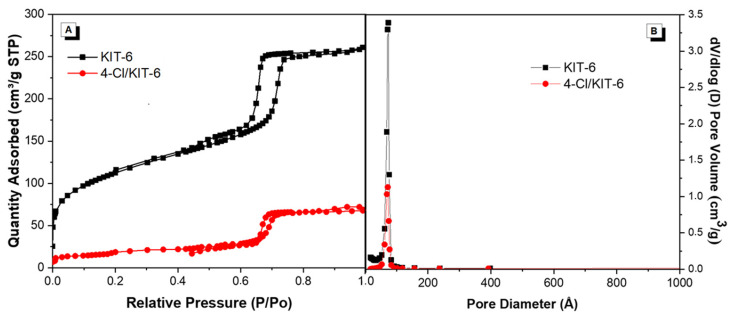
(**A**) N_2_ adsorption/desorption isotherms of KIT-6 (■) and 4-Cl/KIT-6 (●); (**B**) Pore volume distribution of KIT-6 and 4-Cl/KIT-6.

Figure 5 presents the N_2_ adsorption/desorption isotherms of the KIT-6 and 4-Cl/KIT-6 samples, highlighting the mesoporous behavior of the materials and the changes in the hysteresis profile after chalcone encapsulation. The textural properties of the materials are presented in Table 3.

**Figure 6:**
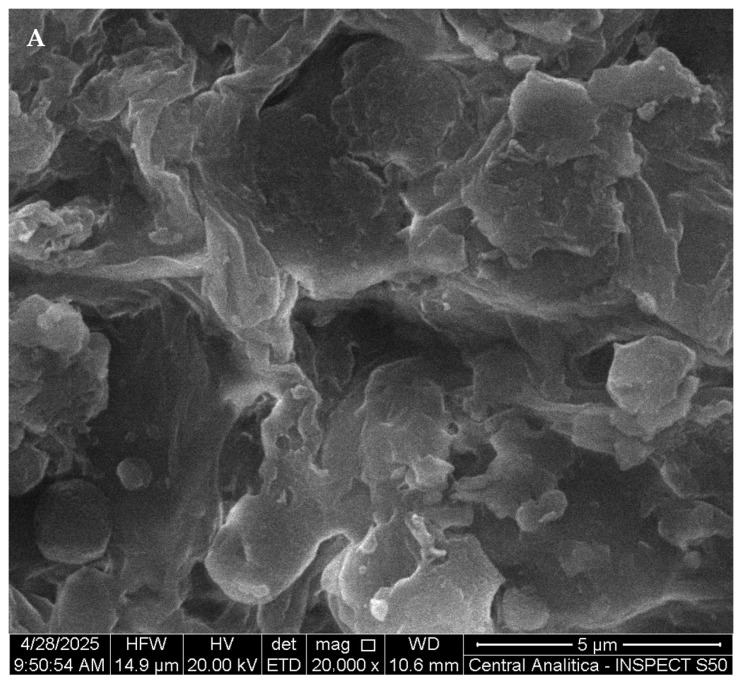

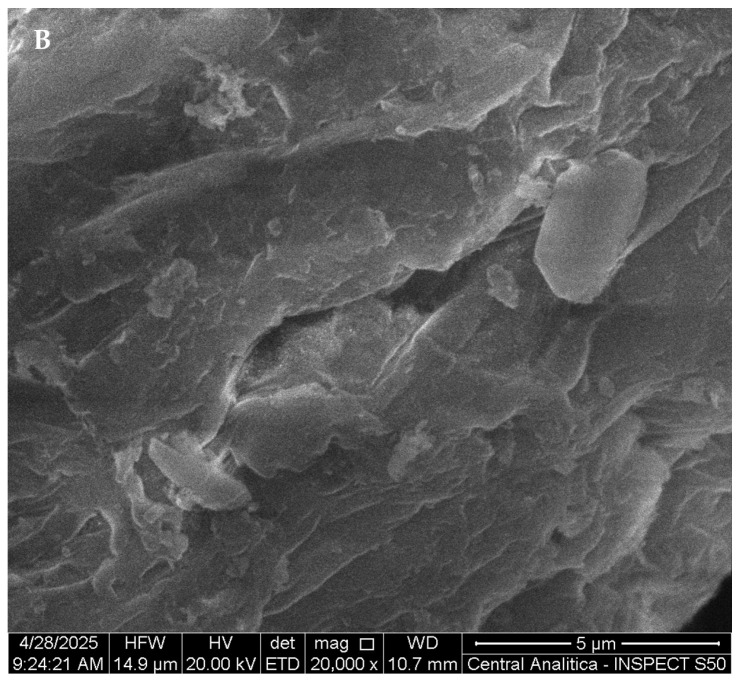
SEM images. KIT-6 (**A**) and 4-Cl/KIT-6 (**B**).

Figure 6 shows scanning electron microscopy (SEM) micrographs obtained for the KIT-6 material (Figure 6A) and after the encapsulation process of chalcone 4-Cl (Figure 6B), analyzed at a magnification of 20,000×.

Moreover, in order to assess the stability of the encapsulating agent (KIT-6), the chalcone (4-Cl), and the complex (4-Cl/KIT-6), zeta potential measurements were performed for the samples. The results are presented in Table 4.

According to the zeta potential analysis, all samples exhibited a negative surface charge in the solvent, with the 4-Cl/KIT-6 complex displaying greater numerical stability. The reduction in negative values observed in the 4-Cl/KIT-6 system can be attributed both to the intrinsically negative charge of KIT-6 and to the presence of chlorine groups in the chalcone structure, which are highly electronegative. It is noteworthy that the mesoporous structures of both KIT-6 and 4-Cl/KIT-6 are considered stable [37], because zeta potential values greater than +30 mV or less than −30 mV are sufficient to prevent particle aggregation and coalescence, thereby ensuring colloidal stability. These findings reinforce the potential of the 4-Cl/KIT-6 complex for pharmacological applications.

### 3.3. In Vitro Cytotoxicity Assessment

**Figure 7:**
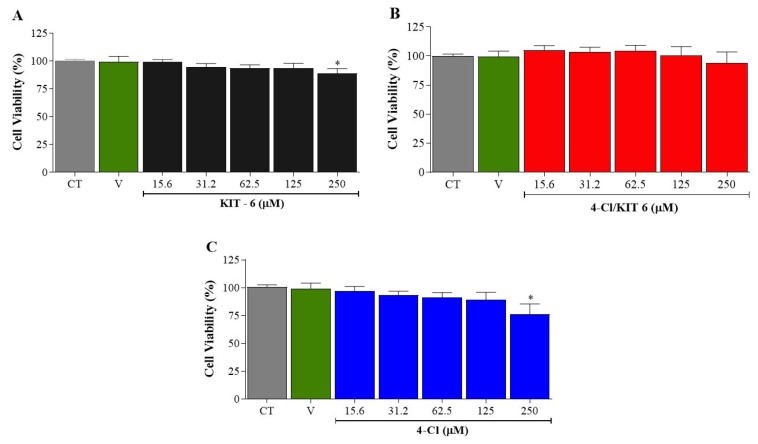
Cytotoxic effects of (**A**) KIT-6, (**B**) 4-Cl/KIT-6, and (**C**) free 4-Cl in PC12 cells, assessed via MTT assay after 24 h of exposure. *X*-axis: sample concentration (µM); *Y*-axis: cell viability (% of control). CT: untreated control; V: vehicle control (0.5% DMSO). Data are presented as mean ± SEM (*n* = 3). Statistical analysis was performed using one-way ANOVA followed by Dunnett’s post hoc test. * *p* < 0.05 vs. CT.

Figure 7 presents the results of the cell viability assessment obtained through the MTT reduction assay performed on the PC12 cell line (rat adrenal medulla pheochromocytoma cells—ATCC CRL-1721). The results showed that KIT-6 and 4-Cl demonstrated significant cytotoxicity at the highest concentration evaluated (250 µm), as illustrated in Figure 7C. On the other hand, the 4-Cl/KIT-6 system did not present cytotoxicity at any of the concentrations tested.

Considering the absence of relevant cytotoxicity in tests with PC12 cells, the studies were expanded to evaluate acute toxicity in vivo, using zebrafish (*Danio rerio*), with the aim of confirming the safety of KIT-6, the 4-Cl/KIT-6 system, and the free compound 4-Cl in a complete biological model.

### 3.4. Toxicological Assessment

Acute toxicity assessment of 4-Cl, 4-Cl/KIT-6, and KIT-6 revealed a favorable safety profile. Doses of 4, 20, and 40 mg/kg (20 μL) of all samples did not result in mortality during the 96 h period, indicating that the LD_50_ is greater than 40 mg/kg.

### 3.5. Formalin-Induced Nociceptive Behavior

**Figure 8:**
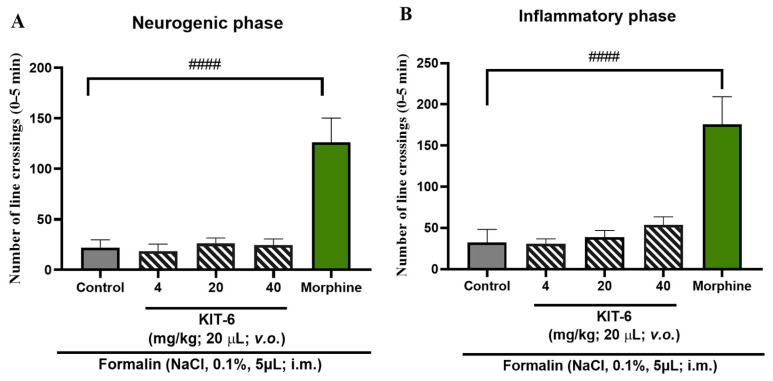
Evaluation of KIT-6 on formalin-induced nociceptive behavior in adult zebrafish, assessed during the (**A**) neurogenic phase (0–5 min) and (**B**) inflammatory phase (15–30 min). Data are presented as mean ± standard error of the mean (*n* = 6 per group). Nociception was triggered by intramuscular injection of 5 µL of 0.1% formalin into the caudal region. Morphine (100 mg/kg; 20 µL; i.p.) was used as the positive control, and the vehicle group received 3% DMSO (20 µL; i.p.). Statistical analysis was performed using one-way ANOVA followed by Tukey’s post hoc test (^####^ *p* < 0.0001 vs. morphine).

Data analysis indicated that KIT-6, when administered alone, was not able to significantly attenuate any of the phases of the formalin-induced nociceptive response, presenting a statistically significant difference in relation to the positive control group (morphine; ^####^ *p* > 0.0001; Figure 8A,B).

**Figure 9:**
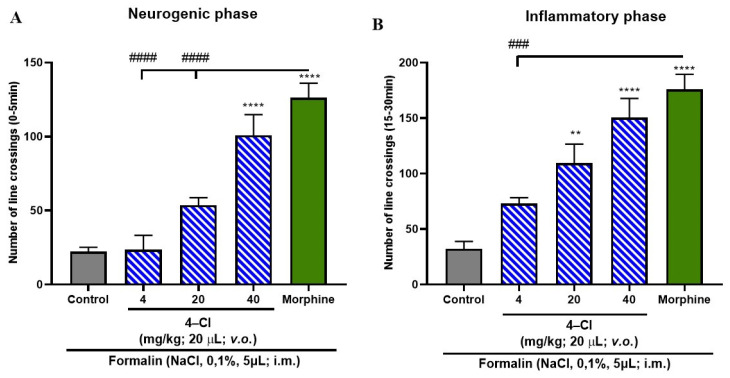
Evaluation of the antinociceptive effect of the free 4-Cl compound in adult zebrafish sub-jected to formalin-induced nociception, with assessments conducted during the (**A**) neurogenic phase (0–5 min) and (**B**) inflammatory phase (15–30 min). Data are presented as mean ± standard error of the mean (*n* = 6 per group). The nociceptive response was elicited by intramuscular injection of 5 µL of 0.1% formalin into the tail. Morphine (100 mg/kg; 20 µL; i.p.) was used as the positive control, while the control group received vehicle (3% DMSO; 20 µL; i.p.). Statistical significance was determined using one-way ANOVA followed by Tukey’s post hoc test (** *p* < 0.01, **** *p* < 0.0001 vs. control; ^###^ *p* < 0.001, ^####^ *p* < 0.0001 vs. morphine).

The free form of the compound 4-Cl exhibited a statistically significant antinociceptive effect, reversing nociceptive behavior in the neurogenic phase only at the 40 mg/kg dose, and in the inflammatory phase at both 20 and 40 mg/kg (*p* < 0.0001 vs. control; Figure 9A,B). Given these findings, the encapsulated form, 4-Cl/KIT-6, was subsequently tested to compare its efficacy profile and assess whether encapsulation could maintain or enhance the compound’s analgesic properties.

Notably, the analgesic effect of the encapsulated system was superior to that observed with the 4-Cl compound in free form (Figure 9A,B), indicating that encapsulation in KIT-6 not only preserved but also enhanced the efficacy of 4-Cl. These data suggest that the 4-Cl/KIT-6 formulation optimizes the bioavailability and action of the compound in both phases of pain, reinforcing the potential of the system as a promising strategy for controlled release with extended therapeutic action.

**Figure 10:**
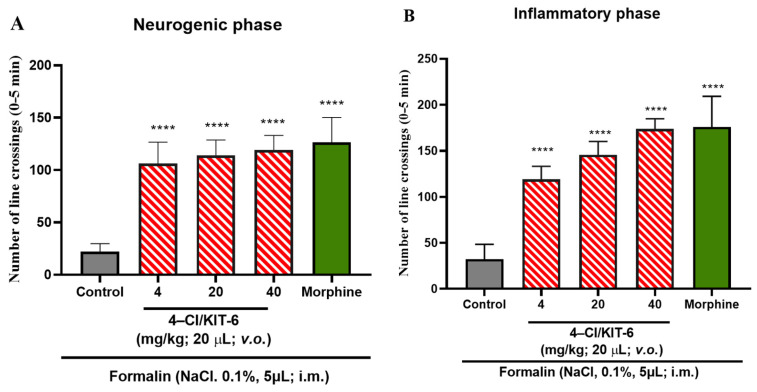
Effect of the 4-Cl/KIT-6 system on formalin-induced nociception in adult zebrafish, analyzed during the (**A**) neurogenic phase (0–5 min) and (**B**) inflammatory phase (15–30 min). Results expressed as mean values ± standard errors of the mean (*n* = 6/group). Nociception was induced by injection of 0.1% formalin (5 µL; i.m.) into the tail. Morphine (100 mg/Kg; 20 µL; i.p.). Control: vehicle (3% DMSO; 20 µL, i.p.). One-way ANOVA followed by Tukey’s test: (**** *p* < 0.0001 vs. control).

As can be seen in the graphs in Figure 10A,B the 4-Cl/KIT-6 system demonstrated significant antinociceptive activity, promoting a significant reduction in the nociceptive response in both the neurogenic and inflammatory phases of the formalin test at all doses analyzed (*p* > 0.0001 vs. control). Since the 4-Cl/KIT-6 system was able to reverse the late phase of inflammatory pain—usually associated with the release of inflammatory mediators and central and peripheral sensitization—its potential anti-inflammatory effect was investigated. In general, substances capable of inhibiting the second phase of pain, as in the formalin test, often show anti-inflammatory activity in experimental models [37]. The second phase is characterized by an inflammatory process involving the release of prostaglandins, cytokines such as TNF-α and IL-1β, and other mediators [38,39]. Research shows that natural and synthetic compounds that reduce this phase are also effective in models of edema, peritonitis, and chronic inflammation, suggesting a strong correlation with anti-inflammatory mechanisms [40,41]. Thus, the reversal of the nociceptive effect by the 4-Cl/KIT-6 system and by 4-Cl in free form on the second phase justifies its evaluation in models of induced inflammation.

### 3.6. Anti-Inflammatory Effect of 4-Cl and 4-Cl/KIT-6 System

**Figure 11:**
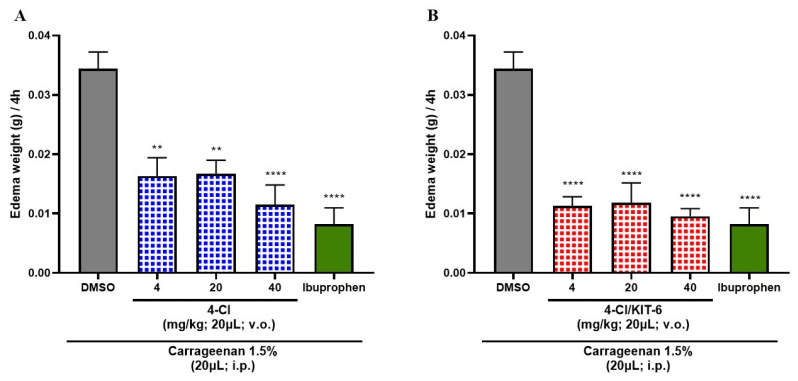
Effect of 4-Cl (**A**) and 4-Cl/KIT-6 system (**B**) on carrageenan-induced abdominal edema in adult zebrafish after 4 h of analysis. Each column represents a mean ± standard error of the mean (*n* = 6/fish). One-way ANOVA, followed by Tukey test: (** *p* < 0.01, **** *p* < 0.0001 vs. control).

According to the data presented in Figure 11, the three tested doses of the 4-Cl sample (Figure 11A) and the 4-Cl/KIT-6 system (Figure 11B) promoted a significant reduction in the inflammatory response compared to the negative control group (3% DMSO), used as a sample dilution vehicle. Both treatments exhibited statistically similar effects to those observed in the group treated with ibuprofen, the reference drug used as a positive control in the assay. However, it is observed that the 4-Cl/KIT-6 system outperformed the 4-Cl compound alone, since all tested doses of the system resulted in more robust statistical differences (***** *p* < 0.0001 vs. control), suggesting greater anti-inflammatory efficacy. These findings indicate that the incorporation of 4-Cl into the KIT-6 mesoporous matrix potentiated its activity, promoting an even more significant anti-inflammatory response, with potency comparable to that of the reference nonsteroidal anti-inflammatory drug.

## 4. Discussion

### 4.1. Characterization

The low-angle X-ray diffraction peaks show three Miller indices characteristic of the mesoporous structure of KIT-6, namely (211), (220), and (332), indicating an ordered pore distribution and confirming the three-dimensional cubic symmetry with space group *Ia3d* [24]. After the encapsulation of 4-Cl, a reduction in peak intensity and a slight shift in the main peak position were observed. This behavior is common in the incorporation of organic compounds into mesoporous materials and is related to the occupation of internal channels, without significantly compromising the mesoporous structural organization of KIT-6.

The high-angle X-ray diffraction pattern of the pure and encapsulated chalcone exhibits intense peaks at 2θ = 16.8°, 18.6°, 24.5°, 25.5°, 27.4°, and 28.2°, indicating a highly crystalline structure. After encapsulation in KIT-6, a decrease in intensity and broadening of the reflections is observed, indicating partial loss of crystallinity—a behavior also reported for chalcones encapsulated in mesoporous silicas, as described by Sayed et al. [30].

For the KIT-6 sample, characteristic absorption bands attributed to the mesoporous silica structure were identified. The bands observed at 1075, 806, and 445 cm^−1^ correspond to the asymmetric and symmetric stretching of Si–O–Si bonds and the bending vibration of the Si–O bond, respectively—features typical of the three-dimensional framework of KIT-6 [42]. These bands confirm the formation of the organized inorganic silica structure and the preservation of its structural integrity.

In the spectrum of 4-chloro-chalcone, bands were identified at 1600 and 1500 cm^−1^, attributed to the stretching vibrations of the C=C double bonds in conjugated aromatic rings. Additionally, the band at 1081 cm^−1^ may be related to vibrations of the substituted aromatic ring, while the bands at 980 and 818 cm^−1^ are associated with out-of-plane deformations of the aromatic rings and the presence of the C–Cl bond, characteristic of substituted aromatic halides [30].

For the hybrid material 4-chloro/KIT-6, the spectrum revealed bands at 1600 and 1500 cm^−1^ from the encapsulated chalcone, in addition to the bands at 1075, 806, and 445 cm^−1^ from the silica matrix. The simultaneous presence of bands from both components confirms the physical incorporation of the chalcone into the KIT-6 structure. The preservation of band positions indicates that no significant chemical modification of the molecule occurred during the encapsulation process. The reduction in the relative intensity of the chalcone’s aromatic bands may be associated with physical interactions with silanol groups on the silica surface, as also observed by Sayed et al. [30] in similar materials such as SBA-15 and MCM-41, suggesting possible hydrogen bond formation and accommodation of the compound within the pores of KIT-6.

Thermogravimetric analysis (TGA/DTG) and differential scanning calorimetry (DSC) of the samples are shown in Figure 4. The corresponding mass loss values from these analyses are summarized in Table 2. The KIT-6 sample (Figure 4A) exhibits a single mass loss event of approximately 4% up to 132 °C, related to the release of physisorbed water. No other thermal events are observed, which is consistent with the inorganic nature of the material, previously calcined, and corroborated by the high residual mass of 96%. Additionally, the DSC profile (Figure 4D) did not reveal significant thermal transitions, confirming its thermal stability.

The 4-Cl chalcone (Figure 4B) presents two decomposition events. The first, predominant one occurs between 25 and 331 °C, with a mass loss of 95%, attributed to the degradation of the organic compound. The second stage, between 331 and 408 °C, accounts for the remaining 5% of the decomposition. The final residue is nonexistent, indicating complete volatilization of the compound. The DSC profile (Figure 4E) shows a broad endothermic peak around 160 °C, possibly related to melting or molecular reorganization, followed by exothermic events between 300 and 450 °C, confirming the TGA data. This thermal behavior aligns with previous studies on structurally similar chalcones [43].

Based on the thermogravimetric analysis (TGA) presented in Table 2, the 4-Cl/KIT-6 sample exhibited a mass loss of 34% in the temperature range of 90 to 343 °C, attributed to the thermal decomposition of the encapsulated 4-Cl compound. From this value, the encapsulation efficiency (EE%) and drug loading capacity (DL%) were estimated using the equations below:

Drug loading (DL%) = (mass of encapsulated drug/total mass of 4-Cl/KIT-6) × 100(1)

Encapsulation efficiency (EE%) = (mass of encapsulated drug/initial mass of drug used) × 100(2)

Considering the initial 1:1 (*w*/*w*) ratio between 4-Cl and KIT-6 used during the preparation, both calculated values were equivalent. These results indicate good incorporation efficiency of the bioactive compound into the mesoporous matrix. The high initial surface area, the structural features of KIT-6, and the presence of silanol groups (Si–OH) capable of interacting with functional groups of 4-Cl may have favored efficient drug adsorption. The DSC (Figure 4F) profile was similar to that of the free chalcone, although with peaks of lower intensity, which may indicate changes in molecular organization and interactions with KIT-6.

Figure 4 shows the N_2_ adsorption/desorption isotherms of the KIT-6 and 4-Cl/KIT-6 samples. Both exhibit type IV(a) isotherms with H1-type hysteresis loops, which are typical of highly ordered mesoporous materials such as KIT-6, and indicative of a uniform pore size distribution [44,45]. The reduction in adsorption volume observed for the 4-Cl/KIT-6 sample suggests decreased pore accessibility, possibly associated with the incorporation of chalcone into the mesoporous structure, in agreement with the structural data obtained by XRD.

As shown in Table 3, the incorporation of chalcone led to a significant reduction in specific surface area and total pore volume. This behavior indicates that the organic molecule is located within the mesopores of KIT-6, partially occupying the channels and limiting access to the internal surface. Moreover, an increase in the average pore diameter is observed after encapsulation, suggesting that the chalcone may be preferentially located in the smaller mesopores, shifting the pore size distribution toward larger diameters and contributing to the reduction of the total pore volume, as described by Thommes et al. [45]. The preservation of the isotherm profile and the H1-type hysteresis confirms that the mesoporous structure remains intact after encapsulation.

Figure 6A shows that the KIT-6 mesoporous silica exhibits a rough surface morphology with visible porosity. After encapsulation (Figure 6B), the surface becomes denser and smoother, with little visible porosity. These changes suggest that the pores were effectively filled by the compound, implying a successful encapsulation predominantly within the internal structure, without external crystalline aggregates.

### 4.2. Cytotoxicity

The results presented in Figure 7 demonstrate that both KIT-6 and the free compound 4-Cl exhibit significant cytotoxicity at a concentration of 250 µM in PC12 cells, as revealed by the MTT assay (Figure 7C). In contrast, the 4-Cl/KIT-6delivery system was free of cytotoxic effects at all concentrations tested.

This differentiation in toxicity is consistent with the literature on mesoporous silica nanoparticles (MSNs). Studies with MSNs in PC12 and other neuronal lines report that, in isolation, these structures present high biocompatibility at concentrations ranging from 1.95 to 1000 µg/mL in periods of up to 24 h, with maintained cell viability, stable ROS levels and no DNA damage [46]. In our study, isolated KIT-6 showed cytotoxicity, suggesting that its formulation—possibly with reduced size or non-functionalized cargo—may have intensified cellular internalization or interaction with endocytic pathways, affecting cellular responses. This is in agreement with a review that highlights that, despite high reactive surface, the toxicity of MSNs depends on factors such as shape, size, porosity, and surface modifications [47].

The 4-Cl/KIT-6 system showed cellular safety at all concentrations. This is because controlled delivery protects against potential mitochondrial disruption. For example, NAD^+^-loaded MSNs maintain ATP and reduce LDH release in PC12 under oxidative stress, demonstrating a mitoprotective effect [48]. This study exemplifies how controlled release can mitigate the toxic impact of active compounds.

The cytotoxicity observed for free 4-Cl at a concentration of 250 µm is compatible with the profile of highly lipophilic aromatic compounds and enonic conjugates [49,50]. Finally, the disparity between the cytotoxic properties of isolated KIT-6 and 4-Cl/KIT-6 shows that the formulation may have altered relevant parameters—such as dispersion, aggregation, bioavailability, and controlled release—thus providing a safer and more effective solution for therapeutic applications involving PC12 and possibly other cell models.

### 4.3. Acute Toxicity 4-Cl, KIT-6 and 4-Cl/KIT-6

Toxicity studies in zebrafish are used as initial screenings for the safety of new molecules, due to the high correlation with mammalian models and the sensitivity of the locomotor system to neurotoxic effects [51,52].

In the present study, the 4-Cl sample demonstrated limited toxicity at the doses tested, without significantly compromising the survival or locomotor activity of the animals, suggesting an acceptable safety profile under experimental conditions. The incorporation strategy has been reported in the literature as an effective approach to improve the pharmacological and toxicological profile of bioactive compounds [53,54].

The 4-Cl/KIT-6 system showed remarkable cellular safety. By incorporating 4-Cl into the mesoporous matrix, there was possible modulation in the release rate and intracellular access to the active compound, reducing local concentration peaks and mitigating mitochondrial stress—a mechanism suggested in studies focusing on slow release as a key factor for toxicological safety [55].

### 4.4. Formalin-Induced Antinociceptive Effect

The obtained data indicate that isolated KIT-6 did not exhibit relevant antinociceptive efficacy in any phase of the formalin-induced pain response, unlike morphine, which served as the positive control. The free 4-Cl compound, in turn, demonstrated the ability to alleviate pain only during the neurogenic phase, and only at the highest dose of 40 mg/kg; in the inflammatory phase, it significantly reduced nociception at doses of 20 mg/kg and 40 mg/kg. Notably, the 4-Cl/KIT-6 combination revealed a more consistent antinociceptive effect, promoting a significant reduction in the pain response in both phases of the test and at all administered doses.

The reversal of the antinociceptive effect observed with the free 4-Cl compound is consistent with earlier reports in the literature. Derivatives of dibenzylideneacetone, par-ticularly those featuring a conjugated dienone structure, have shown notable pain-relieving properties across multiple preclinical pain models, such as the formalin assay and acetic acid-induced writhing test in rodents. These molecules share structural characteristics with curcuminoids, especially due to the α,β-unsaturated moiety, which enables interaction with molecular targets that regulate pain perception [56,57]. These data support the therapeutic potential of 4-Cl as an agent that reduces pain sensitivity, corroborating the findings in animal studies and indicating that its effect may involve the suppression of mediators that facilitate nociceptive signaling [58].

The results of the antinociceptive effect in the 4-Cl/KIT-6 system were significant at all doses of both phases. It is reported in the literature that the incorporation of antinociceptive drugs in mesoporous silica nanocarriers amplifies their activity in the formalin test 58. One study demonstrated that encapsulation of indomethacin in MSNs reduced phase 1 and 2 pain by 53% and 79%, respectively, outperforming the free compound as observed in this study; in addition, it avoided gastric side effects common to the isolated drug [59]

### 4.5. Analysis of the Anti-Inflammatory Effects of 4-Cl and Its Controlled Release via KIT-6

The results obtained demonstrate that both the 4-Cl compound and the 4-Cl/KIT-6 system were able to significantly reduce the inflammatory response compared to the negative control group (DMSO 3%), evidencing the anti-inflammatory potential of both treatments. The statistical similarity between the groups treated with the samples and the positive control group (ibuprofen) reinforces the pharmacological efficacy of 4-Cl, both in its free and transported form. However, it is noteworthy that the 4-Cl/KIT-6 system presented a superior performance to the free compound, suggesting that the incorporation of the drug into the mesoporous matrix can enhance its therapeutic effects. This result may be attributed to a modulated and sustained release potentially promoted by the KIT-6 matrix, which could allow for enhanced drug retention at the inflammatory site and reduced systemic toxicity. However, specific drug release studies are necessary to confirm this effect. Studies with ibuprofen encapsulated in MCM 41 demonstrate that approximately 90% of the drug is gradually released in the first 2 h, maintaining a therapeutic effect without compromising the COX 1 inhibitory activity. Furthermore, in pain models, mesoporous silica loaded with ropivacaine demonstrated a significant increase in sensory block time—from approximately 2 to 6 h—with reduced tissue toxicity, demonstrating the benefits of sustained release [60].

## 5. Conclusions

This study demonstrated that mesoporous silica KIT-6 is an effective delivery system for the encapsulation of the chalcone 4-Cl. Structural, thermal, and textural analyses confirmed the successful incorporation of the compound into the mesoporous matrix without compromising the structural integrity of the silica. XRD and FTIR results showed that the ordered mesoporous structure of KIT-6 was preserved, allowing for the physical incorporation of the chalcone. In vivo assays using zebrafish showed that the 4-Cl/KIT-6 system maintained the desired antinociceptive and anti-inflammatory effects, while exhibiting lower toxicity compared to the free compound. These findings suggest that delivering the active molecule within a mesoporous matrix may help modulate its bioavailability, reducing systemic concentration peaks and, consequently, toxicity. Overall, the results highlight the potential of the 4-Cl/KIT-6 system not only as a promising antinociceptive and anti-inflammatory agent, but also as a formulation with an improved safety profile—an essential feature for progress into preclinical and clinical stages of pharmaceutical development.

## Figures and Tables

**Table 1:** ^1^H and ^13^C NMR data of chalcone 4-Cl in CDCl_3_. The δC and δH chemical shifts are reported in ppm.

C	δ_C_	δ_H_
1	133.2	
2/6	129.3	7.40 (d, J = 8.0 Hz)
3/5	129.5	7.55 (d, J = 8.0 Hz)
4	136.5	
C_α_	125.7	7.04 (d, J = 15.9 Hz)
C_β_	142.0	7.69 (d, J = 15.9 Hz)
C=O	188.3	

**Table 2:** Quantitative mass loss values obtained from TGA/DTGA thermal decomposition curves.

Samples	Mass Loss (%)				
	I	II	I	II	Residue
KIT-6	25–132	-	4	-	96
4-Cl	25–331	331–408	95	5	0
4-Cl/KIT-6	25–90	90–343	5	34	61

**Table 3:** Summary of textural properties.

Sample	S_BET_ (m^2^/g) ^a^	V_T_ (cm^3^/g) ^b^	d_P_ (nm) ^c^
KIT-6	404	0.403	5.38
4-Cl/KIT-6	60	0.111	7.07

^a^ S_BET_ = Specific surface area calculated by the BET method; ^b^ V_T_ = Total pore volume calculated by the BHJ method in *p*/*p*_0_ = 0.98; ^c^ d_P_ = Pore diameter calculated by the BHJ method.

**Table 4:** Summary of zeta potential analysis.

Sample	Zeta Potential (mV)
4-Cl	−20.3 ± 0.98
KIT-6	−35.3 ± 2.30
4-Cl/KIT-6	−44.0 ± 0.71

## Data Availability

The original contributions presented in this study are included in the article. Further inquiries can be directed to the corresponding author(s).
